# Production of membrane proteins for characterisation of their pheromone-sensing and antimicrobial resistance functions

**DOI:** 10.1007/s00249-018-1325-z

**Published:** 2018-07-31

**Authors:** Aalishaa A. Azam, Jean M. Kinder, G. Nasir Khan, Ade Alase, Pikyee Ma, Yang Liu, James R. Ault, Peter J. F. Henderson, Babur Z. Chowdhry, Bruce D. Alexander, Stephen E. Harding, Mary K. Phillips-Jones

**Affiliations:** 1 AMR Biophysics Group, School of Pharmacy and Biomedical Sciences, University of Central Lancashire, Preston, Lancashire PR1 2HE UK; 2 Astbury Centre for Structural Molecular Biology, Faculty of Biological Sciences, University of Leeds, Leeds, LS2 9JT UK; 3 Department of Pharmaceutical, Chemical and Environmental Sciences, University of Greenwich, Central Avenue, Chatham, Kent ME4 4TB UK; 4 National Centre for Macromolecular Hydrodynamics, School of Biosciences, University of Nottingham, Sutton Bonington, Loughborough, Leicestershire LE12 5RD UK

**Keywords:** Histidine kinase, Membrane proteins, Vancomycin resistance, Enterococci, Pheromone sensors, *Streptococcus pneumoniae*, Circular dichroism spectroscopy, Analytical ultracentrifugation

## Abstract

Despite the importance of membrane proteins in cellular processes, studies of these hydrophobic proteins present major technical challenges, including expression and purification for structural and biophysical studies. A modified strategy of that proposed previously by Saidijam et al. (2005) and others, for the routine expression of bacterial membrane proteins involved in environmental sensing and antimicrobial resistance (AMR), is proposed which results in purification of sufficient proteins for biophysical experiments. We report expression successes amongst a collection of enterococcal vancomycin resistance membrane proteins: VanT_G_, VanT_G_-M transporter domain, VanZ and the previously characterised VanS (A-type) histidine protein kinase (HPK). Using the same strategy, we report on the successful amplification and purification of intact BlpH and ComD2 HPKs of *Streptococcus pneumoniae*. Near-UV circular dichroism revealed both recombinant proteins bound their pheromone ligands BlpC and CSP2. Interestingly, CSP1 also interacted with ComD. Finally, we evaluate the alternative strategy for studying sensory HPKs involving isolated soluble sensory domain fragments, exemplified by successful production of VicK_ESD_ of *Enterococcus faecalis* VicK. Purified VicK_ESD_ possessed secondary structure post-purification. Thermal denaturation experiments using far-UV CD, a technique which can be revealing regarding ligand binding, revealed that: (a) VicK_ESD_ denaturation occurs between 15 and 50 °C; and (b) reducing conditions did not detectably affect denaturation profiles suggesting reducing conditions per se are not directly sensed by VicK_ESD_. Our findings provide information on a modified strategy for the successful expression, production and/or storage of bacterial membrane HPKs, AMR proteins and sensory domains for their future crystallisation, and ligand binding studies.

## Introduction

Membrane proteins are crucially important for the physiological functioning of biological cells, possessing roles in molecular recognition and signal transduction, transport of essential nutrients, exclusion of toxic molecules, ion regulation and energy generation. Between 20 and 30% of proteins encoded by eubacterial, archaeon and eukaryotic genomes are membrane proteins (Wallin and von Heijne 1998), and in humans defects in their proper folding or other mutations can result in many serious diseases. Approximately, 60% of current small molecule drugs target membrane proteins (Terstappen and Reggiani 2001; Davey 2004; Arinaminpathy et al. 2009), testifying to the potential of identifying further membrane proteins as new drug targets in the future. Knowledge of the three-dimensional structure of membrane proteins coupled with how they function is one important area for drug discovery goals. But largely due to their hydrophobicity, membrane proteins pose technical challenges for their expression and purification and for structural work such as crystallisation. As a result, only approximately 2% of protein structures in the Protein Data Bank (PDB) are membrane proteins (http://blanco.biomol.uci.edu/mpstruc/; Miles and Wallace 2016).

Yet, there have been recent advances in methods for the production of low abundance membrane proteins, including bacterial membrane proteins (e.g. Potter et al. 2002; and reviewed in Saidijam et al. 2003, 2005; Suzuki and Henderson 2006; Moraes et al. 2014; Hardy et al. 2016; Hussain et al. 2016; Lee et al. 2016; Lee and Pollock 2016; Rawlings 2016), leading to increased successes in crystallisation, structural determination and in elucidation of mechanisms of action (e.g. Weyand et al. 2008; Shimamura et al. 2010; Simmons et al. 2014). Many of the expression and purification strategies for bacterial membrane proteins are based on work with highly hydrophobic membrane transport proteins (Ward et al. 1999; Saidijam et al. 2005; Suzuki and Henderson 2006). Here, we present an adapted strategy based on these established methods for reliable production of membrane histidine protein kinases (HPKs) of bacterial two-component signal transduction systems (Potter et al. 2002; Ma et al. 2008) and a variety of other membrane proteins involved in antimicrobial resistances (AMR) and peptide pheromone sensing, described below.

Resistance by bacteria to current antibiotic agents is recognised to be a major global problem in the treatment of hospital and other infections. Resistance mechanisms (which can be considered as nanomachines) mounted by bacteria in response to exposure to antibiotic drugs often comprise cascades of molecular mechanisms for drug recognition, signal transduction and production of efflux and/or deactivation processes to remove the antibiotic threat and ensure bacterial survival (Phillips-Jones & Harding 2018). Many of the components involved in antibacterial resistances are membrane proteins. Structural elucidation of these components and characterisation of their mechanisms of action are important for future intervention strategies. Biophysical approaches also provide important information. For example, we recently used hydrodynamic methods to demonstrate binding by the glycopeptide antibiotic vancomycin to the VanA-type VanS histidine kinase membrane receptor (Phillips-Jones et al. 2017a, b). Furthermore, using circular dichroism spectroscopy, we showed that glycopeptide binding to VanS is weak (Hughes et al. 2017). Intact VanS protein was produced for these experiments through the commonly used method of heterologous expression of a plasmid-borne gene (in this case *vanS*) in a heterologous *Escherichia coli* host, using a membrane protein overexpression plasmid such as pTTQ18His (e.g. Saidijam et al. 2005). However, modifications to these published strategies for expression and purification were necessary to ensure success. Furthermore, following VanS purification, reduced or no detergent was added for subsequent biophysical experiments to prevent potential inhibition of dimerization and phosphorylation activities, as commonly observed for many HPK proteins. Here, we evaluate these modifications that proved successful for production of active intact VanS, for the expression of a collection of HPK and AMR membrane proteins of different membrane topologies, and for two specific examples of peptide pheromone-sensing HPKs (BlpH and ComD of *Streptococcus pneumoniae* (de Saizieu et al. 2000; Pestova et al. 1996; Cheng et al. 1997; Podbielski & Kreikemeyer 2004), including their ligand binding abilities post-purification. We compare this adapted strategy with that used previously for other *Enterococcus faecalis* HPKs by Ma et al. (2008), who reported the successful expression in *E. coli* of 15 out of 16 of the genome complement of membrane HPK genes and the purification of 12 out of the 15 expressed HPKs. We also investigate another strategy for investigations of ligand binding and sensing mechanisms using only the predicted sensory domain in isolation; we sought to determine whether the soluble extracellular sensory domain of the *Enterococcus faecalis* VicK histidine kinase, VicK_ESD_, lacking its transmembrane segments can be successfully expressed and purified for future characterisation using biophysical techniques.

## Materials and methods

### Gene cloning

#### Genes involved in conferring resistance to glycopeptide antibiotics in enterococci

Plasmids were constructed, sequenced and kindly provided by Dr Djalal Meziane-Cherif and Professor Patrice Courvalin (Pasteur Institute, France). The *Enterococcus faecalis* BM4518 *vanT*_*G*_ (encoding VanG-type serine racemase involved in transport and conversion of l-serine to d-serine) (AAQ16274.1) and *vanT*_*G*_-*M* (encoding residues 1–342 of VanT_G_—the membrane serine transporter component of VanT_G_) (Meziane-Cherif et al. 2015) were cloned into the membrane protein overexpression plasmid pTTQ18His as described in Ma et al. (2008). Cloning and expression of the gene encoding the VanA-type VanS membrane histidine kinase of *E. faecium* B4147 has been described previously (Phillips-Jones et al. 2017a). The VanA-type *vanZ* gene of *E. faecium* B4147 (CDD 121006; AAA65959 Arthur et al. 1995), which confers teicoplanin resistance, was cloned into pMR2, a derivative of pTTQ18His, introducing a His_6_ tag at the N-terminus of the expressed recombinant protein ensuring a predicted location for the His_6_ tag on the inside of the membrane (Rahman et al. 2007).

#### *Streptococcus pneumonia*e ComD and BlpH pheromone-sensing histidine protein kinases

The *blpH* (de Saizieu et al. 2000) and *comD* (Cheng et al. 1997; Pestova et al. 1996) genes of *S. pneumoniae* ATCC700669 were amplified by PCR and cloned into pTTQ18His as described previously (Ma et al. 2008). The *comD* gene was cloned as a *Sac*I-*Sal*I ended fragment, whilst the *blpH* gene was cloned as an *Eco*RI-*Pst*I-ended fragment. Clones were verified by gene sequencing. An RGS(H)_6_ sequence was introduced at the C terminus of both proteins to facilitate purification by nickel affinity methods (Ma et al. 2008).

#### Extracellular sensing domain of enterococcal VicK

The putative extracellular sensing domain of VicK, VicK_ESD_ (equivalent to residues 35–176 of *E. faecalis* V583 *vicK*), was amplified by PCR and cloned as an *Nde*I-*BamH*I fragment into pET14b. The correct sequence was verified by DNA sequencing. The expressed protein with an N-terminal GSS(H)_6_SSGLVPRGSHMI sequence was predicted to be 18,238 Da and this was confirmed by mass spectrometry.

#### Expression studies

All expression plasmids were transformed into *E. coli* BL21 [DE3].

For small-scale expression trials of vancomycin resistance genes, cultures (50 ml) were grown aerobically in Luria–Bertani (LB) broth containing 100 μg ml^−1^ carbenicillin at 37 °C until an absorbance (A_600_) of 0.5 was reached when 1 mM isopropyl *β*-d-1-thiogalactoside (IPTG) was added for induction of *van* gene expression (Ward et al. 1999; Saidijam et al. 2005). Growth was permitted to continue for a further 3 h post-induction at a reduced temperature of 30 °C before cell harvesting. No optimisations of expression conditions were undertaken. Mixed *E. coli* membranes were prepared by the water lysis method (Ward et al. 1999) and samples analysed by SDS-polyacrylamide gel electrophoresis and Western blotting (see below).

Expression of *S. pneumoniae comD* and *blpH* was undertaken in large-scale (6 l) culture experiments employing the non-optimised strategy described above. Briefly, *E. coli* BL21 [DE3] cells harbouring pTTQ*comD* or pTTQ*blpH* were cultured aerobically in 6 l of selective Luria–Bertani (LB) broth at 37 °C and induced with 1 mM IPTG. The cultures were incubated for a further 3 h at a reduced temperature of 30 °C prior to cell harvesting. Cells were lysed by explosive decompression and mixed membranes prepared as described by Ma et al. (2008).

Expression trials of soluble VicK_ESD_ was undertaken by cultivation of *E. coli* BL21 [DE3]/pET14b-VicK_ESD_ in 500 ml of LB broth containing 100 μg.ml^−1^ ampicillin at 37 °C and induction with 0.4 mM IPTG induction as described above for the Van proteins. After incubation for a further 3 h at 37 °C, cells were lysed by sonication and the insoluble membrane fraction harvested by centrifugation at 100,000*g* for 40 min at 4 °C. Separated soluble and insoluble fractions were stored at − 20 °C prior to SDS-PAGE analysis.

All growth experiments included control cultures in the absence of IPTG (uninduced cultures).

#### Purification of BlpH, ComD and VicK_ESD_ proteins

For BlpH and ComD membrane proteins, total mixed membranes of *E. coli* BL21 [DE3] harbouring pTTQ*blpH* or pTTQ*comD* were prepared using methods described previously (Potter et al. 2002; Ma et al. 2008), except that the sucrose density gradient centrifugation step used to separate the inner and outer membranes was omitted. His-tagged BlpH and ComD proteins were solubilised from mixed membranes using 1% (w/v) DDM detergent and purified by nickel affinity chromatography as described by Ma et al. (2008) using wash buffers containing 20 mM imidazole and elution buffers containing 200 mM imidazole, both of which contained 0.05% *n*-dodecyl-*β*-d-maltoside (DDM) (Saidijam et al. 2005). Purified proteins were exchanged into 10 mM sodium phosphate, pH 7.2, containing 0.05% DDM.

For purification of VicK_ESD_ protein from soluble cell fractions, nickel affinity chromatography was undertaken in the absence of DDM, but otherwise according to Ma et al. (2008) using wash buffers containing 20 mM imidazole and elution buffer comprising 200 mM sodium acetate, pH 4.0. Purified protein was exchanged into 20 mM Tris–HCl, pH 7.9, or 20 mM sodium acetate, pH 4.0, or 20 mM sodium phosphate, pH 7.2, or 10 mM ammonium hydrogen carbonate, pH 8.0, using Centricon filter devices.

#### SDS-polyacrylamide gel electrophoresis (SDS-PAGE)

Proteins were separated by SDS-PAGE using 4–5% stacking and 12% resolving SDS-polyacrylamide gels prepared and subjected to electrophoresis according to Sambrook et al. (1989). For Western blotting experiments, the separated proteins were first electroblotted onto Fluorotrans Transfer membrane (Pall Corp.) as described previously (Ma et al. 2008).

#### Western blotting

Western blotting experiments to detect the presence of the His_6_ tags of recombinant overexpressed proteins were performed using the INDIA™ HisProbe-HRP (Perbio Science UK Ltd) with Supersignal West Pico Chemiluminescent substrate (Pierce) detection as described previously (Ma et al. 2008).

#### Mass spectrometry

To confirm the molecular masses of purified VicK_ESD_ and VanS, the electrospray ionisation mass spectrometry facility of the Astbury Centre for Structural Molecular Biology, University of Leeds, UK, was used (Phillips-Jones et al. 2017a).

#### N-terminal sequencing

Purified proteins (VanS and VicK_ESD_) were transferred to Fluorotrans Transfer membrane (Pall Corp.), visualised by Coomassie Blue G-250 staining and bands excised for sequencing using Edman degradation (Alta Biosciences, University of Birmingham, UK).

#### Protein determination

Protein determinations were carried out using bicinchoninic acid in a Pierce™ BCA Protein Assay Kit (Thermo Scientific) according to the manufacturer’s instructions. Bovine serum albumin was used as standard.

#### BlpH and ComD ligands

Mature BlpC, CSP1 and CSP2 peptide ligands were synthesised and verified by Cambridge Peptides (Birmingham, UK) and dissolved in 100% acetonitrile.

**Table:** 

Peptide	
BlpC	*N*-GLWEDLLYNINRYAHYIT-C
ComC CSP1	*N*-EMRLSKFFRDFILQRKK-C
ComC CSP2	*N*-EMRISRIILDFLFLRKK-C

#### Circular dichroism spectroscopy (CD)

CD experiments were performed using a nitrogen-flushed Jasco J715 spectropolarimeter (for VicK_ESD_) or an Applied Photophysics Chirascan Plus instrument (for ComD and BlpH). Purified membrane proteins were typically prepared in 10 mM sodium phosphate, pH 7.2–7.6 (in the presence of 0.05% DDM for membrane proteins) (Patching et al. 2012; Miles and Wallace 2016) and allowed to equilibrate for 20 min at 15 or 20 °C prior to acquisition of spectral data. A bandwidth of 2 nm was used and data acquired at 1 s/nm. No data in which the HT of the detector exceeded 600 V were included in the analyses.

For CD measurements in the far-UV (180–260 nm), protein concentrations in the range 2.4–14.0 μM (~ 0.12–0.25 mg ml^−1^) were employed in sample volumes of 200–350 μl using a pathlength of 1 mm. The longer pathlength cell was selected on the basis of the buffer and detergent concentrations used here (Miles and Wallace 2016). For VicK_ESD_ measurements at 15 and 37 °C, 30 scans were obtained; for thermal denaturation experiments, 5 scans were obtained. For BlpH and ComD proteins, spectra are the average of two scans. Thermal denaturation experiments for VicK_ESD_ were performed at a starting temperature of 15 °C which was increased stepwise and incrementally to 90 °C; at each step, 10 min equilibration time was permitted before acquisition of spectral data.

For CD measurements in the near-UV region (250–340 nm), protein concentrations of 3.6 µM (0.2 mg ml^−1^) (ComD) or 9.5 µM (BlpH) were employed in sample volumes of 400 µl using a pathlength of 10 mm. Spectra shown are the average of ten scans. Reaction mixes were incubated with peptide ligands (or equivalent volumes of acetonitrile solvent) for 20 min prior to acquisition of spectral data as described by Patching et al. (2012).

In all cases, control spectra of buffers with other relevant additives (in the absence of added proteins) were also obtained. These spectra were subtracted from spectra obtained for each purified protein and used to derive difference spectra. The maximum concentration of acetonitrile in ligand experiments was 0.45%.

## Results

### Expression of VanS, VanT_G_, VanT_G_-M and VanZ membrane proteins involved in vancomycin resistance

**Fig. 1:**
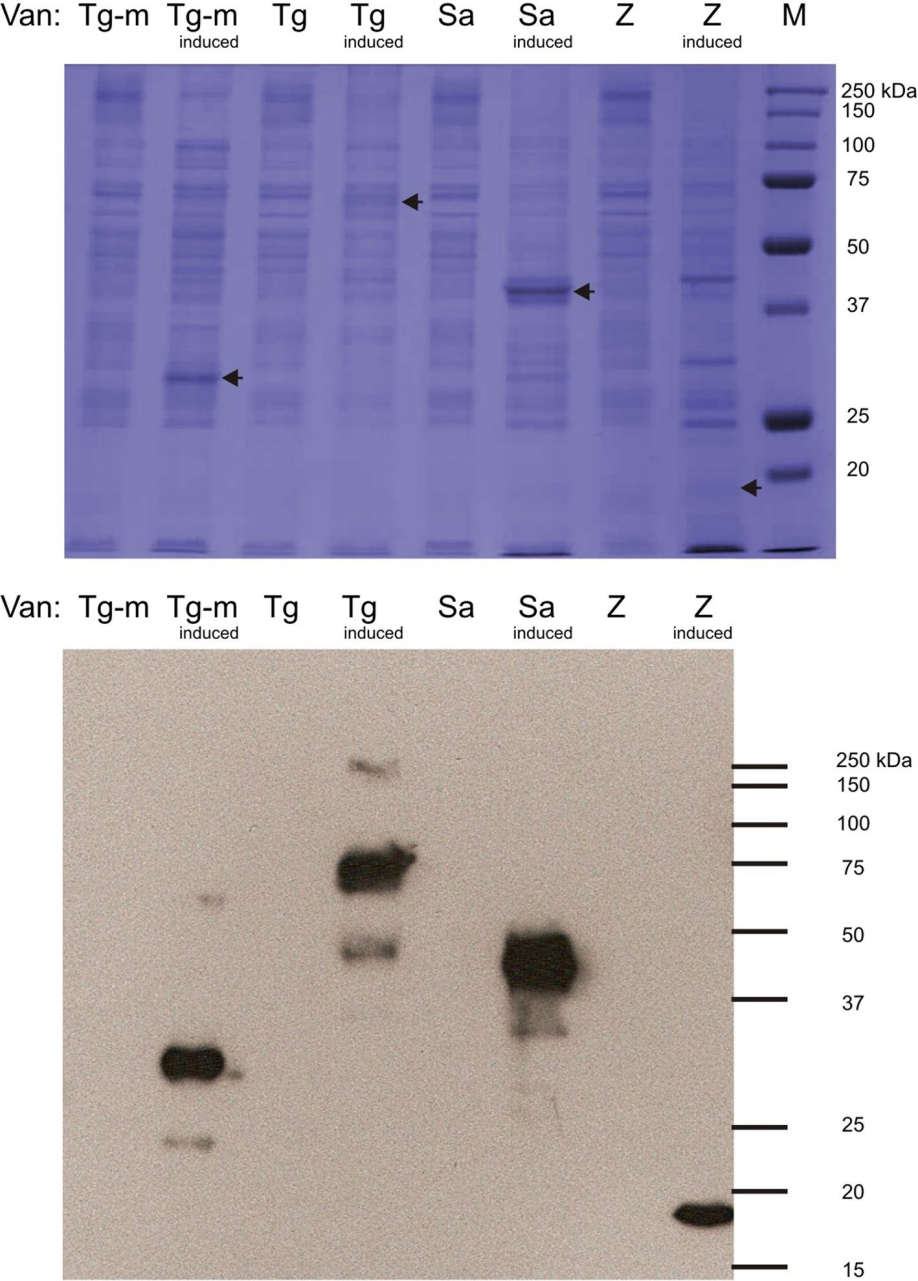
Expression in *E. coli* BL21 [DE3] mixed membranes of intact His_6_-tagged vancomycin resistance proteins: VanT_G_ (serine racemace) of *E. faecalis* BM4518, VanT_G_-M (residues 1–342; a truncated version of native VanT_G_ encoding only the putative serine membrane transporter portion), VanS_A_ and VanZ_A_ of *E. faecium* B4147. Mixed membranes (10 μg) from cultures harbouring pTTQ-*vanT*_*G*_, pTTQ-*vanT*_*G*_-*M*, pTTQ-*vanS*_*A*_ (Phillips-Jones et al. 2017a) or pMR2-*vanZ* (Rahman et al. 2007) expression plasmids and induced with 1 mM iso-propylthiogalactoside (IPTG) or uninduced cultures carrying the same plasmids were loaded on SDS-polyacrylamide gels (4% stacking/12% resolving). Following electrophoresis, gels were either stained with Coomassie Brilliant Blue for visual detection of protein bands (uppermost panel) or proteins were transferred electrophoretically onto PVDF membrane for Western blotting using an INDIA His probe followed by exposure to photographic film (lowermost panel)

Genes encoding enterococcal vancomycin resistance determinants VanT_G_, (a VanG-type serine racemase), VanS_A_ (the VanA-type membrane histidine protein kinase) and VanZ_A_ (a membrane protein conferring teicoplanin resistance) were cloned into the membrane protein overexpression plasmid pTTQ18His (*vanT*_*G*_, *vanS*_*A*_) or its derivative plasmid pMR2 (*vanZ*_*A*_) using the approaches described previously for other membrane proteins (Potter et al. 2002; Ma et al. 2008; Phillips-Jones et al. 2017a). VanZ has an uneven number (five) of predicted transmembrane segments and so its encoding gene was cloned into pMR2, which fuses a His_6_ tag at the N-terminus of the expressed recombinant protein, thereby ensuring that upon expression of VanZ, the His_6_ tag is located intracellularly. VanS and VanT_G_ are predicted to possess two and ten membrane-spanning segments, respectively. In addition, a truncated version of *vanT*_*G*_ was also cloned into pTTQ18His; residues 1–342 of VanT_G_ comprise the serine transporter domain of the full-length protein and hence also possess the ten transmembrane segments of the serine transporter but lack the racemase domain (Meziane-Cherif et al. 2015). The same domain architecture and functions have been shown to occur in homologues of other Van-type species and strains, including VanC-type strains (Arias et al. 1999, 2000, 2003; Reynolds & Courvalin 2005). Sequence-verified plasmids were transformed into *E. coli* BL21 [DE3] which was cultured and induced for *vanT*_*G*_, *vanT*_*G*_-*M*, *vanS* and *vanZ* expression, as described in “Materials and methods”. Figure 1 shows SDS-polyacrylamide gel (SDS-PAGE) and Western blot analysis of mixed membranes prepared from induced and control uninduced cultures. Membranes prepared from induced cultures harbouring each expression plasmid, pTTQ-*vanT*_*G*_, pTTQ-*vanT*_*G*_-*M*, pTTQ-*vanS* and pTTQ-*vanZ*, all showed additional bands which gave a positive reaction in the Western blots with the INDIA His probe for detection of His_6_ tags, indicative of successful expression of all four membrane proteins (Fig. 1b).

Additional bands in induced cultures harbouring pTTQ-*vanT*_*G*_-*M* and pTTQ-*vanS* were also visible in the SDS-PAGE gels (Fig. 1a) indicative of higher levels of overexpression. No signals were detected in any of the uninduced control membranes, indicating tight regulatory control of the p*tac* promoter of pTTQ18His in the absence of inducer, which is consistent with previous observations (Ma et al. 2008). The predicted masses of the His-tagged proteins are 81.2 kDa (VanT_G_), 38.8 kDa (VanT_G_-M), 45.8 kDa (VanS) and 20.1 kDa (VanZ). The positive protein bands shown in Fig. 1 appear of lower mass than these predicted values. This is likely to be due to anomalous migration behaviour that is typical of hydrophobic membrane proteins in the approximate technique of SDS-PAGE. However, final confirmation that the detected protein bands are indeed intact VanT_G_, VanT_G_-M and VanZ would routinely be carried out following purification of each protein via mass spectrometry and/or N-terminal sequencing (see Ma et al. 2008 for example). Indeed, the intact nature of the VanS protein has already been confirmed in our laboratory; VanS was purified as described in Phillips-Jones et al. (2017a) and a mass of 45,775 Da was determined using electrospray ionisation mass spectrometry (ESI-MS) which closely matches the predicted mass of 45,765 Da. Further confirmation was provided by sedimentation equilibrium experiments, which determined the overall weight average molar mass to be 46.4–47.7 kDa (Phillips-Jones et al. 2017a). N-terminal sequencing confirmed the presence of the expected n-MNSHM sequence and the protein was shown to retain its autophosphorylation activity post-purification (Phillips-Jones et al. 2017a).

### Expression, purification and verification of *S. pneumoniae* BlpH and ComD histidine protein kinases

**Fig. 2:**
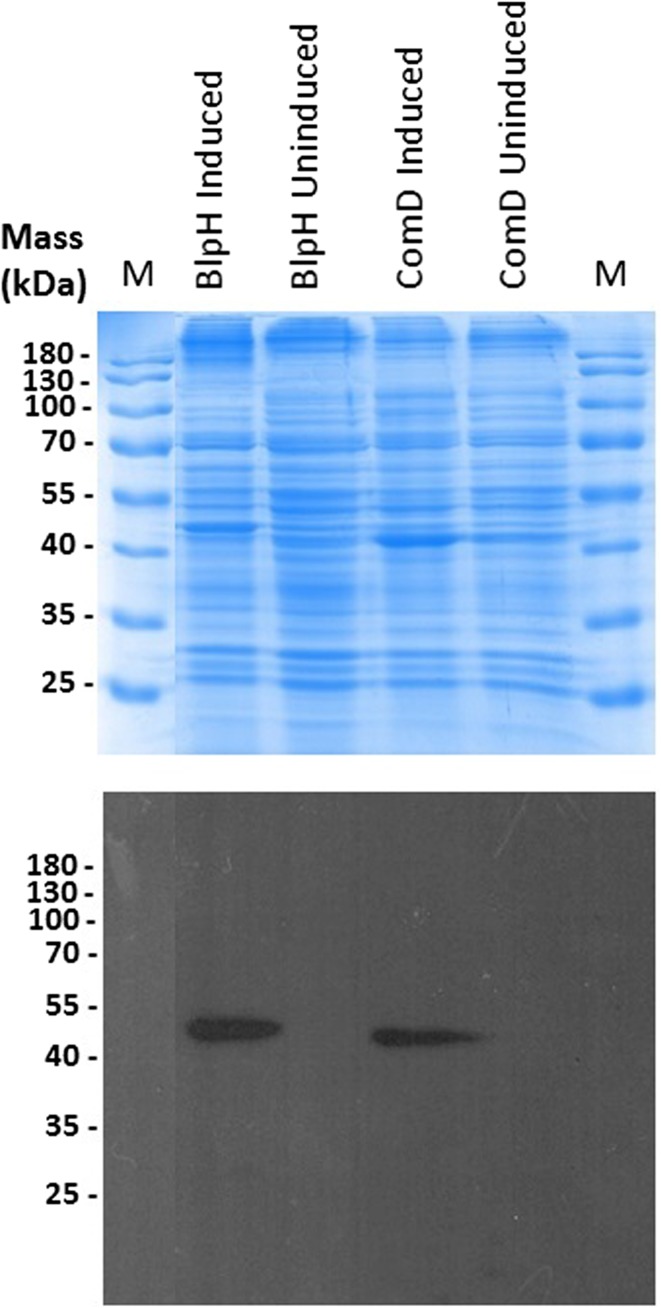
Expression in *E.coli* BL21 [DE3] mixed membranes of intact His_6_-tagged BlpH and ComD membrane histidine kinases of *Streptococcus pneumoniae*. Mixed membranes (20 μg) from cultures harbouring pTTQ*blpH* or pTTQ*comD* and induced with 1 mM iso-propylthiogalactoside (IPTG) or uninduced cultures carrying the same plasmids were loaded on SDS-polyacrylamide gels (4% stacking/12% resolving). Following electrophoresis, gels were either stained with Coomassie Brilliant Blue for visual detection of protein bands (uppermost panel) or proteins were transferred electrophoretically onto PVDF membrane for Western blotting using an INDIA His probe followed by exposure to photographic film (lowermost panel)

**Fig. 3:**
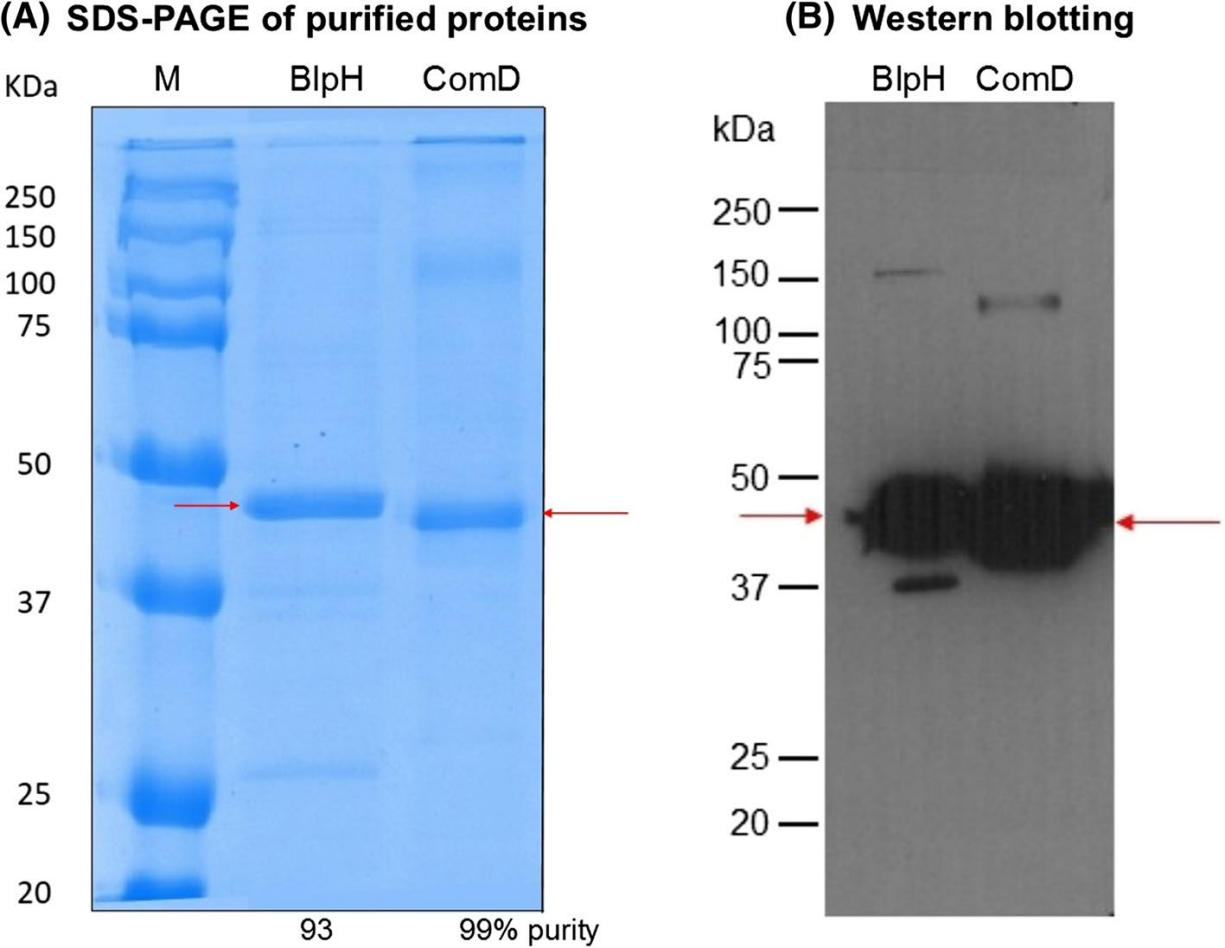
Purification of His_6_-tagged BlpH and ComD histidine kinases from *E. coli* BL21 [DE3] mixed membranes. Nickel affinity methods were used as described in “Materials and methods” using wash buffers containing 20 mM imidazole and elute buffers containing 200 mM imidazole as described in Ma et al. 2008. **a** SDS-PAGE and Coomassie Brilliant Blue staining of purified proteins (4 μg BlpH and 5 μg ComD); % values shown below each lane indicate the % purity of each protein determined by densitometry. **b** Western blot analysis using 5 μg purified protein

Successful expression of intact BlpH and ComD membrane HPKs from *S. pneumoniae* ATCC 700669 was achieved through expression of pTTQ18His-based constructs in *E. coli* BL21 [DE3]. Figure 2 shows samples of mixed membrane preparations of IPTG-induced and -uninduced cultures in which additional bands for both putatively expressed proteins are visible in SDS-polyacrylamide gel analysis of samples derived from induced cultures. The additional bands also give positive signals with the INDIA His probe, confirming the presence of His-tagged proteins. Recombinant His-tagged BlpH and ComD have predicted molecular masses of 53,439 and 52,725 Da, respectively. In common with the Van proteins described here and indeed many other membrane proteins previously, the apparent molecular masses of recombinant BlpH and ComD in SDS-PAGE appear lower than predicted (Fig. 2). Once again, although these proteins require verification that they have remained intact post-purification, it is likely that the smaller apparent masses are attributable to their anomalous migration behaviour in SDS-PAGE as described previously for other hydrophobic membrane proteins (Ma et al. 2008; Phillips-Jones et al. 2017a, b). That the expressed proteins possess intact C-termini was verified by Western blot analysis; positive signals were obtained with the His probe that detects the C-terminal hexa-His motif (Fig. 2). The proteins were successfully purified using the nickel affinity purification protocol described in “Materials and methods” (Fig. 3). Purity was approximately 93–99% as determined by densitometry measurements.

### Ligand binding studies using purified BlpH and ComD

**Fig. 4:**
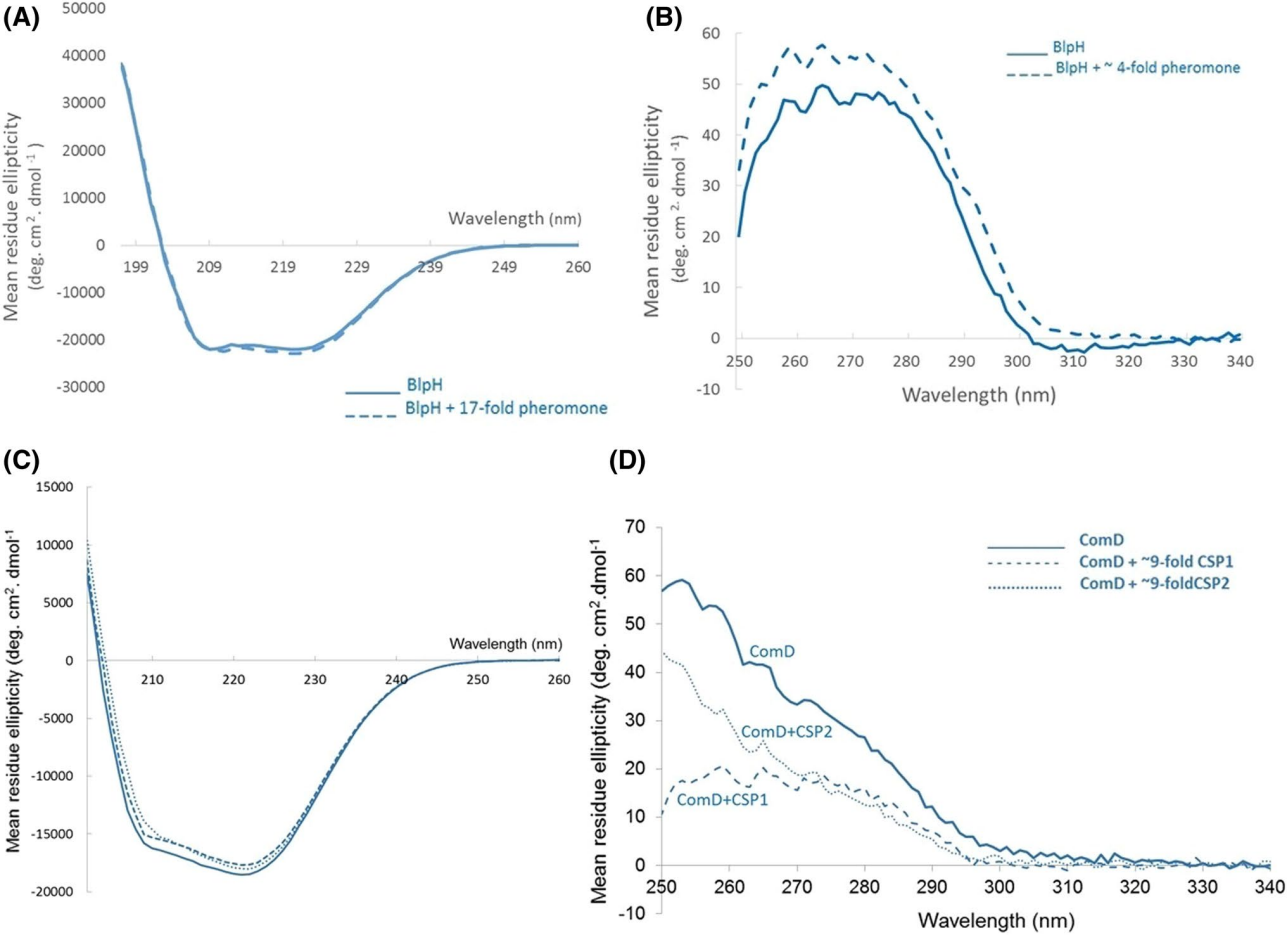
Characterisation of purified membrane histidine kinases BlpH and ComD in 10 mM sodium phosphate pH 7.2 containing 0.05% DDM in the presence and absence of peptide pheromone ligands using CD spectroscopy. Far-UV difference spectra for **a** BlpH (2.4 μM) and **c** ComD (3.6 μM) are shown for wavelength ranges of 197–260 nm (BlpH) or 202–260 nm (ComD) using average data of two repeats measured in a 1 mm pathlength cell, 2 nm bandwidth and 1 s/nm at 20 °C. Spectra were set to zero at 255 nm. Near-UV difference spectra for **b** BlpH (9.5 µM) in the presence and absence of fourfold (38 µM) BlpC peptide pheromone, and **d** ComD (3.6 µM) in the presence and absence of ~ ninefold (~ 32 µM) CSP1 or CSP2 peptide pheromones are shown using averaged data of ten repeat scans measured in a 10 mm pathlength cell, 2 nm bandwidth and 1 s/nm at 20 °C. Spectra were set to zero at 335 nm. Unsmoothed data are shown. For both proteins, difference spectra are shown in which spectral contributions of buffer or other additives have been subtracted

Purified BlpH and ComD were used in far-UV CD spectroscopy experiments to determine whether the purified proteins possessed secondary structure following detergent-based purification. Figure 4a, c shows spectra for His-tagged BlpH and ComD, respectively, obtained in the far-UV region. The spectra of both proteins were typical of those for α-helical membrane proteins, with spectral minima observed in the regions of 208 and 222 nm, confirming possession of structural integrity in both purified BlpH and ComD post-purification (Fig. 4) (Kelly et al. 2005; Patching et al. 2012; Siligardi et al. 2014). The presence of excess synthetic mature BlpC pheromone had no significant effect on the secondary structural conformation of BlpH (Fig. 4a). Similarly, the presence of mature CSP1 or CSP2 exerted no detectable effect on ComD secondary structure (Fig. 4c). However, near-UV difference spectra in the presence of the BlpC pheromone revealed small, but significant spectral changes, indicative of changes in tertiary structural conformation and confirming interactions between BlpH and BlpC. Spectral changes occurred in the 255–270 nm range of Phe side chain transitions as well as the 275–282 nm peaks attributable to Tyr residues, with a less marked change in the 290–305 nm region attributable to Trp. Hence the environments of the side chains of Phe, Tyr and to a lesser extent Trp were all affected during the tertiary structural changes induced by the interaction between BlpH and the BlpC pheromone (Fig. 4b). Tertiary structural conformational change in the presence of peptide pheromone was also demonstrated in the present study for ComD (belonging to pherotype ComD2) (Fig. 4d). Near-UV difference spectra in the presence and absence of peptide pheromone CSP2 [ComC2; the cognate peptide pheromone to which ComD from *S. pneumoniae* ATCC 700669 responds (Croucher et al. 2009)] revealed a significant change in the environments of Phe and to a lesser extent Tyr residues (Fig. 4d), confirming interactions by this known peptide ligand. Interestingly, CSP1 (ComC1) also exerted an effect on the near-UV difference spectrum, indicative of binding. Indeed, the effect of CSP1 on the environment of Phe residues was overall more marked than the effect of CSP2, indicating different tertiary structural changes in response to each peptide (Fig. 4d).

### Cloning, overexpression and purification of VicK_ESD_, the extracellular sensory domain of VicK of *E. faecalis* V583

**Fig. 5:**
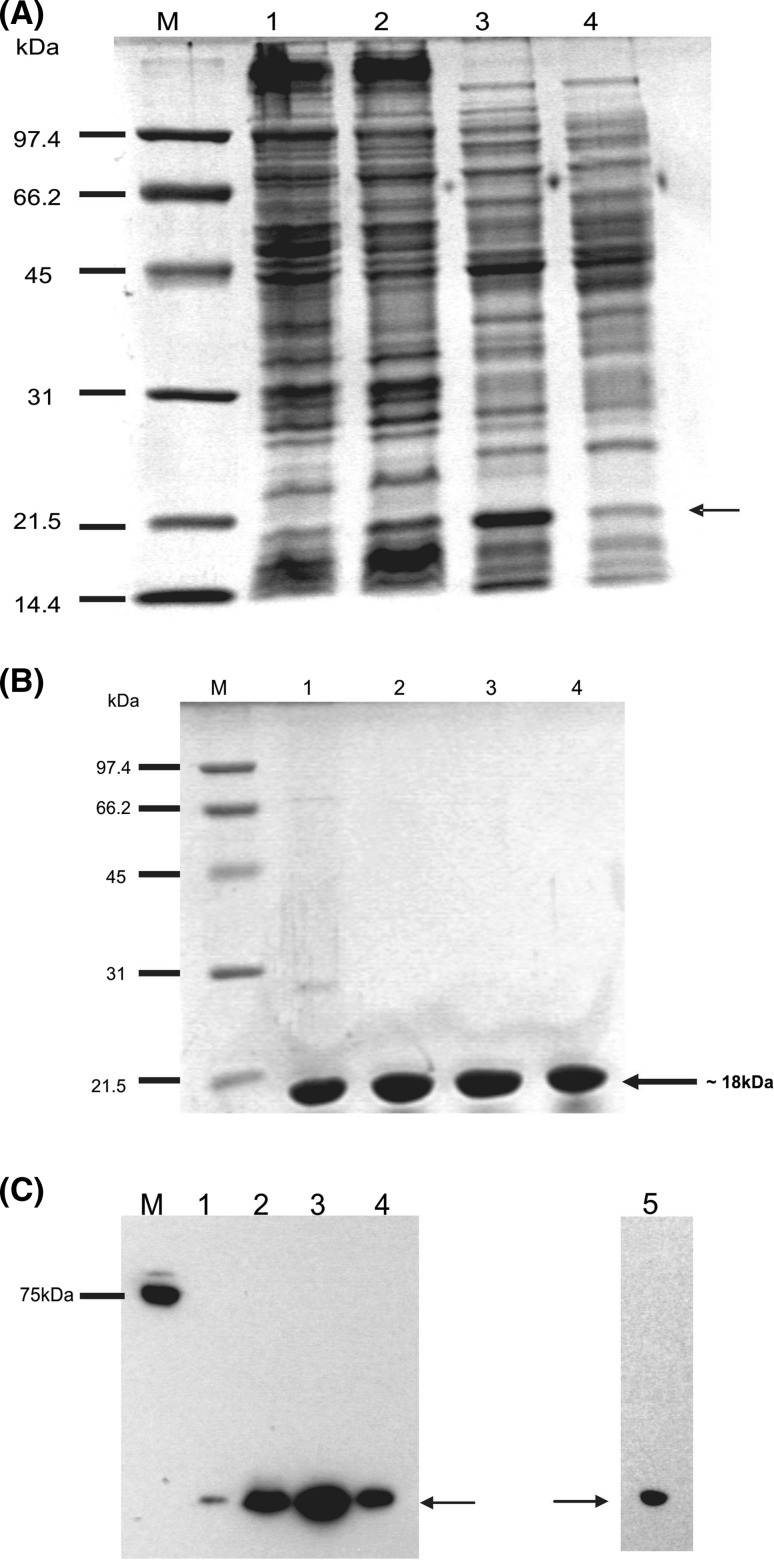
Expression and purification of His_6_-tagged VicK_ESD_. **a** Soluble and insoluble fractions of *E. coli* BL21 [DE3] cells harbouring pET14b-*vicK*_*ESD*_ were prepared by sonication of cell cultures and centrifugation at 100,000*g* for 40 min to sediment-insoluble material. Proteins were resolved using 12% SDS-polyacrylamide resolving gels and visualised with Coomassie Brilliant Blue stain. M: molecular mass markers; lane 1: uninduced insoluble fraction (20 μg); lane 2: induced insoluble fraction (20 μg); lane 3: induced soluble fraction (20 μg); and lane 4: uninduced soluble fraction (20 μg). **b** His_6_-tagged VicK_ESD_ was purified from *E. coli* soluble fractions using nickel affinity methods as described in “Materials and methods”, eluted with 200 mM sodium acetate pH 4.0 and exchanged into lane 1: 20 mM Tris.HCl pH 7.9; lane 2: 20 mM sodium acetate pH 4.0; lane 3: 20 mM sodium phosphate pH 7.2; or lane 4: 10 mM ammonium hydrogen carbonate pH 8.0 using Centricon-10 kDa filter devices; equivalent volumes of final ‘filtered’ protein solutions were analysed by SDS-PAGE and visualised using Coomassie Brilliant Blue staining. **c** Western blot analysis of purified VicK_ESD_, and soluble and insoluble fractions of *E. coli* BL21 [DE3]/pET14b-*vicK*_*ESD*_ lysates (30 μg protein). Proteins were transferred electrophoretically onto PVDF membrane and detected for the presence of a His_6_ tag using an INDIA His probe followed by exposure to photographic film. Lane 1: uninduced insoluble fraction (20 μg); lane 2: induced insoluble fraction (20 μg); lane 3: induced soluble fraction (20 μg); lane 4: uninduced soluble fraction (20 μg); and lane 5: purified VicK_ESD_ (4 μg). M, molecular mass markers (the 75 kDa marker gives a positive signal with the His probe)

An alternative strategy to using full-length membrane sensor kinases to study sensing mechanisms is to use the sensory domains in isolation. To determine whether a truncated soluble version of VicK possessing the predicted sensing domain in isolation could be successfully expressed and purified, the pET14b-VicK_ESD_ construct described in “Materials and methods” was expressed in *E. coli* BL21 [DE3]. Induction of VicK_ESD_ expression was performed through the addition of 0.4 mM IPTG as described in “Materials and methods” and cultures were then incubated for a further 3 h at 37 °C post-induction prior to cell harvesting. Uninduced control cultures achieved similar growth rates as induced cultures indicating that VicK_ESD_ expression is not deleterious to growth of *E. coli* under these conditions (data not shown). Following cell sonication and centrifugation, soluble and insoluble fractions were analysed by SDS-polyacrylamide gel electrophoresis and Western blotting with His probe detection (Fig. 5). Figure 5a shows Coomassie Brilliant Blue-stained SDS-polyacrylamide gels of soluble and insoluble fractions of IPTG-induced and uninduced cultures. Only the soluble fractions from induced cultures showed the presence of an amplified additional visible protein band in SDS-polyacrylamide gels at the expected position of ~ 18 kDa (Fig. 5a). Western blot analysis of these fractions using a His probe to detect the presence of the N-terminal His_6_ tag is shown in Fig. 5c. Although the putative VicK_ESD_ protein was present in all fractions, a greater proportion was found in soluble fractions originating from IPTG-induced cultures (Fig. 5c). It is interesting to note the presence of putative VicK_ESD_ in the uninduced cultures too, possibly indicative of leaky *vicK*_*ESD*_ expression in the absence of inducer using this pET14b expression plasmid. VicK_ESD_ was purified from soluble fractions to at least 90% purity as described in “Materials and methods” and verified by Western blotting (Fig. 5c), N-terminal sequencing (in which the expected correct n-GSSHHHH sequence was obtained) and by mass spectrometry (in which a mass of 18,255 Da was obtained which matches closely the predicted mass of 18,210 Da). Purified VicK_ESD_ was exchanged into four different buffers. Any significant insolubility of purified VicK_ESD_ in any of these buffers was screened through retention of insoluble protein in Centricon filters resulting in reduced protein passing through into the filtrate. Figure 5b shows no detectable difference in VicK_ESD_ levels in the filtrates from all buffers, suggesting that the protein at ~ 19 mg/ml is equally soluble in all four buffers. The successful production of high concentrations of purified VicK_ESD_ is therefore established here.

### Structural status and thermal denaturation studies of VicK_ESD_ investigated using far-UV CD spectroscopy

**Fig. 6:**
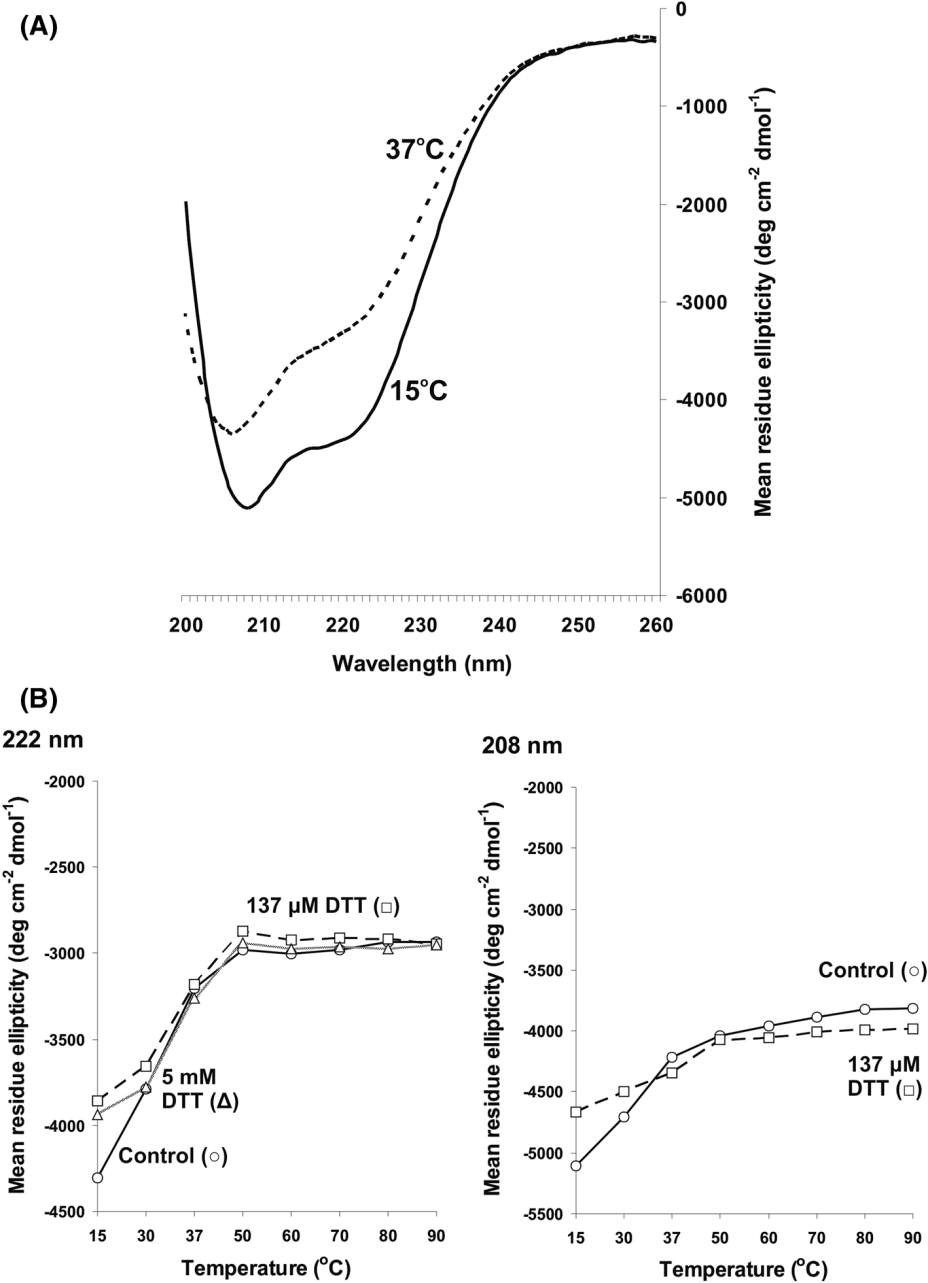
Thermal stability of VicK_ESD_ in the presence and absence of dithiothreitol investigated by CD spectroscopy. Purified VicK_ESD_ (14 μM; 0.25 mg/ml) in 10 mM sodium phosphate, pH 7.6, was used in far-UV measurements using 2 nm bandwidth and 1 s/nm. Temperature ramping was between 15 and 90 °C. **a** For 15 and 37 °C temperature points, 30 repeat scans between 190 and 260 nm were obtained. For comparisons of data obtained at the two temperatures, difference spectra were adjusted to the same values at 260 nm; **b** for other temperature points, five scans were obtained. Measurements were obtained in the presence and absence of the reducing conditions of 137 μM or 5 mM dithiothreitol (DTT). Trough wavelength minima were determined experimentally (data not shown) to be 208 and 222 nm and the spectral data at those minima are shown

To firstly determine whether purified VicK_ESD_ possessed secondary structure and therefore structural integrity post-purification, CD spectroscopy in the far-UV region was undertaken using 0.25 mg/ml protein. Figure 6a reveals difference spectral data (200–260 nm with HT values < 600 V), consistent with that of an α-helical protein with spectra minima at 208 and 222 nm and therefore confirming the presence of secondary structure following the purification process. The difference spectrum at 15 °C is deeper than that obtained at 37 °C, suggesting that VicK_ESD_ exhibits some degree of denaturation through loss in secondary structure during the incremental temperature ramping steps from 15 to 37 C (Fig. 6a). Figure 6b shows the spectral minima values at 208 and 222 nm during the thermal ramping steps. These data suggest that most thermal denaturation occurs predominantly in a temperature range from 15 to 50 °C. Above 50 °C and up to 90 °C, there is little further denaturation and the protein secondary structure is relatively stabilised. The presence of dithiothreitol (DTT)-induced reducing conditions had little effect on the thermal denaturation profiles, though the spectral minima obtained at the initial 15 and 30 °C were shallower than those obtained in the absence of DTT, suggesting that the reducing agent may exert a small destabilising effect on VicK_ESD_ secondary structural conformation in the lower temperature range (Fig. 6b).

## Discussion

**Fig. 7:**
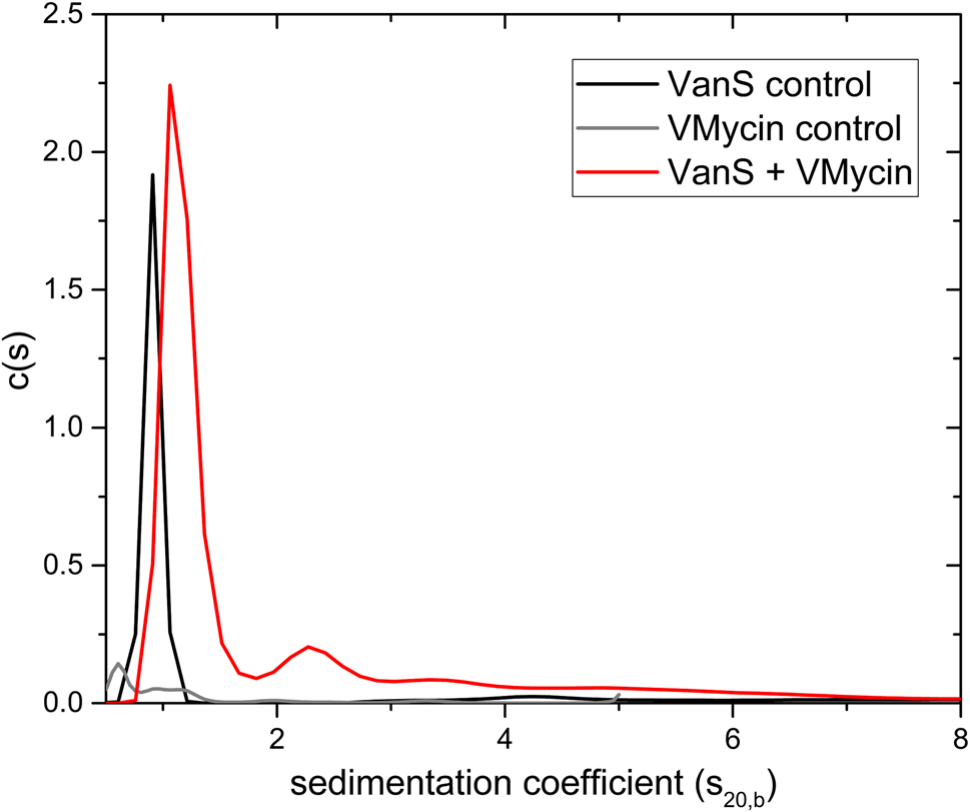
Sedimentation coefficient concentration distribution, *c(s)* vs *s* profile for intact VanS (5.4 μM) (black line) in HGN buffer (containing 20% glycerol) pH ~ 7.9, *I* = 0.1, at 20.0 °C. The rotor speed was 40,000 rpm. The profile for 12.8 μM vancomycin is shown (grey line). VanS and vancomycin is shown by the red line under the same conditions. Reproduced with permission from Phillips-Jones et al. (2017a) Sci Rep 7:46,180

At the 23rd International AUC Workshop & Symposium held at the University of Glasgow in July 2017, we reported the application of hydrodynamic methods using analytical ultracentrifugation (AUC) to characterise the enterococcal VanA-type VanS membrane protein and to explore the possibility that the glycopeptide antibiotic vancomycin binds to VanS directly, resulting in the activation of the VanSR two-component signal transduction system for controlled expression of vancomycin resistance genes. AUC methods have been successfully applied previously for the study of integral membrane proteins and/or their supporting environments and for membrane-exporting proteins with high α-helical content (e.g. Schüler et al. 2000; Inagaki et al. 2013; Le Roy et al. 2015; Surya and Torres 2015; Xu et al. 2015; Lee et al. 2016; Inagaki & Ghirlando 2017; Jaturontakul et al. 2017). We showed direct interactions by vancomycin with purified intact VanS (Fig. 7) (Phillips-Jones et al. 2017a, b), albeit weakly, as confirmed in our later study (Hughes et al. 2017). The hydrodynamics approach also revealed that under the detergentless, membraneless conditions of our experiments, purified VanS remained monomeric even following vancomycin binding. It is possible that to observe downstream changes in VanS following vancomycin binding (such as dimerization), the membrane environment is also required; our studies are now pursuing this possibility. VanS [which possesses only two predicted membrane-spanning regions (TMs)] was expressed using the membrane protein expression plasmid pTTQ18His in *E. coli* BL21 [DE3], also used for most of the other intact membrane proteins in this study. This system was employed here because it proved successful previously for the expression and subsequent purification of a large proportion of other enterococcal HPK membrane proteins (Ma et al. 2008). In the present study, we have established that this same host/plasmid system can also be used to express membrane proteins involved in vancomycin resistance and a truncated version of one of these proteins VanT_G_-M possessing only the N-terminal l-serine membrane transporter domain (and lacking the soluble C-terminal serine racemase domain) (Meziane-Cherif et al. 2015). All four Van resistance membrane proteins investigated here, including the serine racemase VanT_G_ and its truncated version VanT_G_-M, were successfully expressed (Fig. 1). The location of the His_6_ tags in all these recombinant proteins was predicted to be intracellular following the use of either pTTQ18His (which incorporates a C-terminal His_6_ tag) for proteins with an even number of predicted TMs (VanT_G_, VanT_G_-M, VanS) or the use of the related plasmid pMR2 (which introduces an N-terminal His_6_ tag but is otherwise very similar to pTTQ18His) for VanZ, which has an uneven number of five predicted TMs. The adapted strategy described in the present study, which comprises routine use of LB broth for cell culturing, 1 mM inducer and a rapidly reduced post-induction temperature of 30 °C with no further optimisations of these conditions, proved successful, as all proteins were successfully expressed and located to and integrated within *E. coli* membranes. If necessary, growth conditions can be further optimised in future studies to increase the expression of these resistance proteins, but this proved unnecessary in the case of VanS purification for biophysical characterisation (Phillips-Jones et al. 2017a).

To the best of our knowledge, this is the first study to successfully express and purify the intact versions of the *S. pneumoniae* BlpH and ComD (pherotype 2) membrane sensor kinases. A previous study utilised domain fragments lacking the transmembrane domains (Sanchez et al. 2015). The successful expression and purification of these two membrane HPKs (Figs. 2, 3) adds to the high success rate of expressing members of this membrane protein family originating from Gram-positive species in the membranes of a Gram-negative *E. coli* host. A total of 15 out of the 16 membrane HPKs from *E. faecalis* were successfully expressed previously, many of which were expressed using a strategy similar to that proposed here (Ma et al. 2008). In addition, 12 of the 15 expressed proteins were successfully purified (Ma et al. 2008). All purified HPKs tested so far, including ComD and BlpH in the present study, possessed structural integrity post-purification as revealed by far-UV CD spectroscopy (Fig. 4). In addition, purified ComD and BlpH interacted with and bound their peptide pheromone ligands CSP2 and BlpC, as revealed in the near-UV CD difference spectral measurements (Fig. 4). Together with previous successes in ligand binding studies using the same approach with other intact sensor kinases [e.g. FsrC (Patching et al. 2012) and VanS (Phillips-Jones et al. 2017a)], these results further strengthen the promise and potential of the in vitro approach described here for routine identification of ligands and ligand screening methods. Of particular interest is the finding here that the CSP1 pheromone, as well as the native CSP2 ligand, interacted with ComD2 (Fig. 4d). CSP1 and CSP2 belong to two distinct and major pherotypes of *S. pneumoniae* (pherotypes 1 and 2, respectively) and are proposed to exhibit specific interactions only with their cognate ComD HPK of their respective pherotypes, thereby inducing competence for their own specific pherotype only (Pozzi et al. 1996; Iannelli et al. 2005; Johnsborg et al. 2006; Allan et al. 2007; Shpakov 2009). Consistent with that evidence, it is possible that the binding to ComD shown in the present study by both CSPs (which exhibit approximately 50% sequence identity) also occurs in vivo resulting in different conformational change outcomes (consistent with Fig. 4d data), with only that due to CSP2 binding resulting in downstream activation of ComD and ComE and thence induction of competence. However, there are other possibilities. For example, some *S. pneumoniae* strains/pherotypes have been reported to be inducible for competence by both CSP1 and CSP2 (Pozzi et al. 1996), which would suggest that strain ATCC 700669 used here might provide another example of such strains. Alternatively, it has been shown that high concentrations of non-cognate CSP1 can induce competence in pherotype 2 strains in vitro (Iannelli et al. 2005). These possibilities are now being investigated.

The expression strategy described here was therefore successfully applied for reliable expression of the membrane proteins reported here and we suggest that it can be used for expressing other members of these protein families without the need for further optimisations. Out of a total of 21 HPK membrane proteins attempted so far, 20 have been successfully expressed as intact proteins and 17 were successfully purified (Potter et al. 2002; Ma et al. 2008; Phillips-Jones et al. 2017a; and the present study). The adapted strategy is a modification of that originally proposed for bacterial transporter and other membrane proteins (Ward et al. 1999; Saidijam et al. 2003, 2005) and some of the key features are highlighted in the summary below:

1. Batch growth of at least 6 l of *E. coli* BL21 [DE3] host bacterium harbouring pTTQ18His or pMR2 expression plasmids with the cloned HPK gene. In our experience, the most suitable culturing conditions are aerobic culturing in Luria–Bertani (LB) selective broth [without recourse to minimal or very rich media used previously for expression of members of some other membrane protein families, but which in our hands usually produces lower expression levels of HPKs (unpublished data)].
2. At mid-exponential phase, addition of 1 mM isopropyl-thiogalactoside inducer of HPK gene expression, followed by a rapid reduction in incubation temperature to 30 °C. Post-induction incubation is usually of 3 h duration, but may benefit from longer time periods for some proteins. Cell harvesting by centrifugation and preparation of mixed *E. coli* membranes is as described in Ward et al. (1999).
3. Purification of His_6_-tagged HPK proteins is by nickel affinity chromatography as described previously (Ward et al. 1999; Ma et al. 2008) using HEPES-based buffers containing 20% glycerol and 0.05% suitable detergent such as *n*-dodecyl-*β*-d-maltoside (DDM) in the presence of 20 mM imidazole in wash buffers and 200 mM imidazole in elute buffers. Sodium chloride is usually omitted. During buffer exchanges, the detergent concentration is usually reduced to 0.025% which is still above the critical micelle concentration (CMC) value for DDM (0.17 mM or 0.0087%), yet retains the activity of most HPKs and their ability to dimerize upon ligand binding.

For many years, one of our main aims for expressing and purifying intact membrane HPKs, rather than domain fragments or soluble portions lacking the transmembrane regions, has been to investigate ligand (and inhibitor) binding and the ensuing downstream molecular events that then take place including transduction of the ligand signal (or inhibitor signal) across the membrane to the soluble domains within the cytoplasm involved in phosphorylation (Potter et al. 2002, 2006; Ma et al. 2008, 2011; Patching et al. 2012; Phillips-Jones et al. 2013, 2017a, b). The approach seems logical given that the sensory domains are often located within the transmembrane segments. Yet, such an approach applied to membrane sensor kinases is in some cases much more challenging to undertake than for the more hydrophobic membrane transport proteins for which detergent is absolutely essential to maintain solubility and function. Membrane sensor kinases may possess as few as two transmembrane segments with a much larger soluble portion comprising the kinase and ATP-binding domains that reside in the cytoplasm in vivo. The two predicted transmembrane segments of the A-type VanS make up just 10.8% of the protein. Therefore, for functional studies the large soluble portion as well as the ‘minor’ hydrophobic portion of the transmembrane domain must both be considered. Solubility trials often influence how to proceed—for VanS, solubility was often better when detergent was omitted post-elution (Phillips-Jones et al. 2017a). Use of detergent-less buffers facilitated identification by hydrodynamic methods of vancomycin as a ligand; binding was observed in the absence of detergent. Indeed, VanS by itself had a symmetry of 12:1 in aqueous solvent which then became 5:1 in the presence of ligand, suggesting a significant conformational change and possibly behaviour reminiscent of intrinsic disorder proteins in aqueous solution (Dunker et al. 2002). But the accompanying dimerization event was lacking (Phillips-Jones et al. 2017b), though the protein was shown to be active in activity-based buffers (Phillips-Jones et al. 2017a, b). So, perhaps a detergent or a membrane environment is required after all to observe some events, though in our experience the presence of detergent usually reduces or abolishes HPK autophosphorylation activities. In any case, in the example of VanS in the absence of detergent, ligand identification was successfully achieved.

Another approach for investigations of ligand binding by membrane HPKs is to clone and express the predicted sensing domains in isolation, without the TMs. If the sensing domain is sufficiently large, then this eliminates the challenges posed by the hydrophobic full-length membrane proteins and studies can be pursued in the absence of added detergent. It assumes, however, that the TMs are not involved in the sensing mechanism. This approach was successfully used recently in studies of YycG-ex, the extracellular sensing domain of the *S. aureus* VicK homologue, YycG. The crystal structure of YycG-ex was determined and proved insightful for determining how the intact protein functions (Kim et al. 2016). Adopting a similar approach here for *E. faecalis* VicK_ESD_, which possesses a predicted sensing domain of 141 residues, the sensing domain was successfully overexpressed as a recombinant His-tagged soluble protein of 162 residues (lacking the fMet) and approximately 18 kDa (Fig. 5). The purified protein retained structural integrity post-purification and was soluble at concentrations of up to 19 mg/ml suitable for crystallisation and other structural studies. The domain was overall α-helical and exhibited some denaturation upon increases in temperature from 15 to 50 °C, as revealed through measurements of secondary structure composition, but at > 50 °C structural integrity remained relatively stable (Fig. 6).

## Conclusion

For successful and routine expression in *E. coli* of intact bacterial histidine kinase and vancomycin resistance membrane proteins, an adapted strategy of the methods described by Ward et al. (1999) and Saidijam et al. (2005), and described in the present study was shown to be sufficient to obtain the expression of all these membrane proteins. Further optimisations can then be undertaken to maximise membrane protein production, but for the proteins tested here no further optimisations were necessary for obtaining sufficient purified proteins for downstream biophysical characterisations, including identification and confirmation of ligand interactions by ComD and BlpH. Expression of sensing domains in isolation may be another useful approach for producing members of these protein families.

## Abbreviations

AMR: Antimicrobial resistance
HPK: Histidine protein kinase
ESD: Extracellular sensing domain
LB: Luria–Bertani broth
IPTG: Isopropyl β-d-1-thiogalactoside
DDM: *N*-dodecyl β-d-maltoside
